# Annotations

**Published:** 1888-08-25

**Authors:** 


### ANNOTATIONS.

**Ambulances Wanted.**

A FATAL accident occurred on Saturday last, which painfully illustrates and emphasises the need for ambulances in connexion with general hospitals. Lewis McDonald, a young carpenter, was at work repairing one of the upper rooms at the Royal College of Surgeons, Lincoln's Inn, when a plaster ornament attached to the ceiling suddenly gave way, and fell with its full weight upon the unfortunate man's back and head, fracturing his skull and breaking his back. McDonald was at first removed to King's College Hospital; but, after being detained there for a considerable time, it was found there was no vacant bed, and he was thereupon deported to Charing Cross. He died the same evening. In returning a verdict of accidental death, the jury expressed surprise that no ambulance was kept at King's College Hospital. If the jury were surprised that no ambulance is kept at a comparatively small hospital like King's College, what would they have said if they had been told that there is probably no general hospital in London, or indeed in the United Kingdom, which is supplied with an efficient ambulance? The need for ambulance aid is so urgent and so constant, and the difficulty of supplying that need so very inconsiderable, that it is almost impossible to believe such aid is hardly ever available in connexion with a general hospital. The real reason why ambulances are not provided seems to be that it is nobody's business to provide them. The public naturally asks, "Why do the great hospitals not each provide their own?" The answer to that is twofold—first, it would be very costly to provide a separate ambulance and horses for each considerable hospital; and secondly, it is not necessary. What is wanted is that each large town should have one or more ambulances at convenient centres, which should be available for all hospitals; and not only so, but for the removal of sick persons of all classes. This is a question of immediate practical moment, and demands solution. It is everybody's business, and therefore it is nobody's. But those who devote time and thought to hospitals must make up their minds to deal with it. So far as London is concerned, there is a body of philanthropists whose business it would seem especially to be to see that the metropolis is provided with ambulances and central stations. That body is The Hospitals Association. The Association has made a new start, with an energetic secretary, and it should now show itself a practical as well as a deliberative organisation. In these days people have little patience with mere talk. They want to see work, and results. The Hospitals Association is appealing for increased membership and support. Let it prove that it deserves support by some public work of admitted value which everybody can see and take account of. The public will only be too glad in that case to give it all the support it requires.

**The Weather Notwithstanding.**

PEOPLE are beginning to ask the question what is the use of going away to the country or the seaside in such weather as this? It is much better, say they, to stay at home, where you can have good fires, warm underclothing, and plenty of blankets and eider-downs. No doubt that has a show of sense; but people do not go away from London merely to enjoy country pleasures. They go for rest and change. For hardworked Londoners a period of rest and change is as essential as food to the hungry. Not only must it be rest, but it must be of sufficient duration. It is very certain that many of those who are in the full swing of London life, and of course of town life everywhere, habitually work too hard. Where one measures his work strictly by his capacity, ten thousand make no such measurement, but simply tear away as hard as they can until the strength gives out. This way of working may be borne without much very evident injury by men over thirty and under fifty, but it tells very badly upon those who are immature and those who are no longer young. There is no way of avoiding a dangerous breakdown except that of periodical rests. These rests must include change. Indeed, the change is imperative, in order that the rest may be real and complete. Who can find any real rest in London? Who, at least, who is in the full swing of London life? If the worker is in town he must be at his post; and if he is at his post he must work. There is no way of obtaining rest worth the name except that of putting a couple of hundred miles at least between our holiday abode and our work. The evenings and the end of the week spent at the seaside, with the days in London, are a delusion and a snare. It is questionable whether such sham holidays do not do more harm than good. We repeat that a Londoner must have periodical holidays—weeks of complete change and complete rest, and that whatever sort of weather it may be. If it should rain every day, and all day long, and even if there should be snow and ice in August, the hardworked man must make it an absolute duty to be at least four weeks away from his office and his work.

**The Value of a Little Medical Knowledge.**

POMPOUS pedants and self-flattering pharisees are never weary of proclaiming the dangers of a "little knowledge." It is always understood, of course, that the preachers themselves possess a very great deal, and that their knowledge is of the newest and the most valuable kind. An incident which happened last week may help to open the eyes of the infallibles, if indeed their eyes are not like Carlyle's oyster, and persist in remaining obstinately shut. John Crear, an under gardener, of Wylam-on-Tyne, had occupied a portion of his leisure by attending ambulance lectures. He had profited so much that two certificates of proficiency in first aid were given to him. A few days ago he had an opportunity of proving the value of his "little knowledge." A coachman at Mr. Cookson's, of Oakwood, cut his forearm with a hay knife, severing all the principal blood-vessels. A first year's medical student knows that under such circumstances the operation of bleeding to death may only last a few minutes. Crear, however, was on the spot, and with a stick placed over the brachial artery, and a bandage firmly applied over the stick, controlled the bleeding at once and completely. In the meantime, Dr. Foulis was summoned, and expressed his gratification at finding his patient in a condition of perfect safety. In his judgment, Crear had saved the coachman's life. This is an incident of a kind which will be common enough in no long time. It is beginning to be recognised that medicine is for man, and not man for medicine. It should be a source of great satisfaction to every well-disposed person that doctors in all parts of the country are giving their aid to ambulance associations, and that a knowledge of simple life-saving operations such as that practised by Crear is spreading on all hands. This, however, is not enough. Schoolmasters should not only be able to teach elementary physiology, but also such rudimentary surgery as ambulance societies teach. Every schoolboy and schoolgirl in schools of all grades should be taught those facts and methods of procedure which are easy of application by instructed common sense. To make of medicine a mystery in any degree whatever is not only injurious to the public, but suicidal to the medical profession. When will people learn that selfishness, under whatever name it may be hidden, is always and everywhere detestable. Medicine and surgery have everything to gain and absolutely nothing to lose by the fullest proclamation of all their methods and objects, even from the house-tops. This does not mean self medication. Self medication is a delusion because it is an impossibility.

